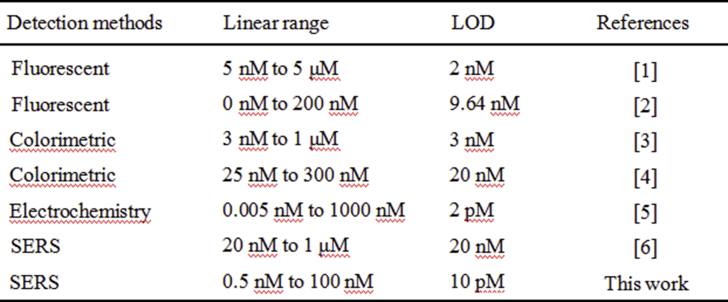# A “turn-off” SERS aptasensor based DNAzyme-gold nanorod for ultrasensitive lead ion detection

**DOI:** 10.1016/j.acax.2019.100020

**Published:** 2019-06-18

**Authors:** Wei Xu, Aiwu Zhao, Fangtao Zuo, Hafiz Muhammad Jafar Hussain, Ranjha Khan

**Affiliations:** a Institute of Intelligent Machines, Chinese Academy of Sciences, Hefei, 230031, People's Republic of China; b Department of Chemistry, University of Science and Technology of China, Hefei, Anhui, 230026, People's Republic of China; c State Key Laboratory of Transducer Technology, Chinese Academy of Sciences, Hefei, 230031, People's Republic of China; d School of Life Sciences, University of Science and Technology of China, Hefei, 230027, People's Republic of China

**Keywords:** Lead (II) ions, Gold nanorods, DNAzyme, Surface-enhanced Raman scattering, Sensitivity, Complex biological samples

## Abstract

It is great significance to precisely monitor lead (II) ions (Pb^2+^) for environment protection and human health monitoring. We designed a sensitive detection strategy for sensitive and selective determination of Pb^2+^, based on a Pb^2+^-specific DNAzyme as the catalytic unit, Cy3-labeled DNA modified gold nanorods (AuNRs) as SERS reporter. Firstly, AuNRs surface were employed as a platform for the immobilization of thiolated probe DNA, and then hybridized with DNAzyme catalytic beacons. By taking advantage of DNAzyme digest, a molecular beacon, causes a “turn-off” SERS signal by disrupting the labeled probes. Under the optical conditions, the DNAzyme-AuNRs sensor system exhibited high sensitivity, acceptable stability and reproducibility with a wide linear range from 0.5 to 100 nM (R^2^ = 0.9973), and an ultra-low detection limit of 0.01 nM. The proposed strategy has additional advantages of being less time-consuming, low-cost and remote query, and avoids the interference of some metals such as Fe^3+^, Cd^2+^, Ba^2+^, Cu^2+^, Zn^2+^. The SERS biosensor system has been successfully applied for detecting Pb^2+^ in real samples with a satisfactory result. The result indicated that the proposed sensing strategy not only enriches SERS platform of monitoring Pb^2+^ but also exhibits potential for the point-of-care diagnostic application of the clinical screening in complicated biological samples.

## Highlights

• A novel SERS aptasensor based on DNAzyme and AuNRs substrate was developed for Pb^2+^ detection.
• Highly selective and sensitive (LOD of 1× 10^-11^ M) for Pb^2+^ detection is performed.
• The proposed strategy was employed to detect Pb^2+^ with high sensitivity and good accuracy in real complex samples.
• The SERS aptasensor exhibited excellent sensitivity, high selectivity, and good reproducibility.

## Graphical abstract

**Figure:**
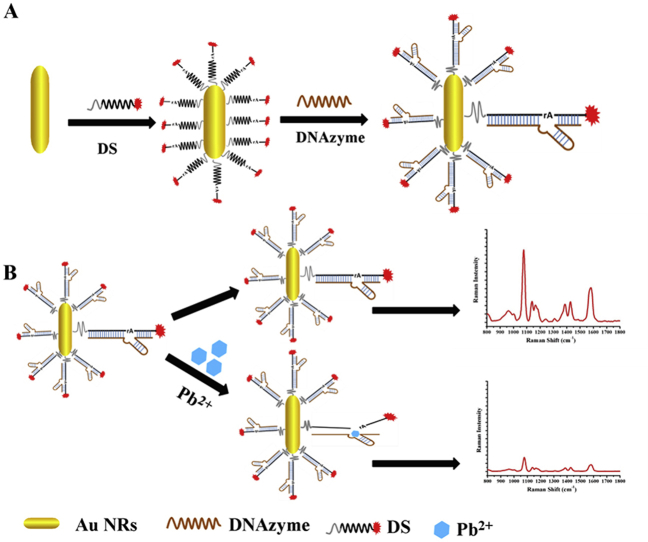

Lead and its compounds, one kind of widespread pollutant, have drawn significant attention in recent decades due to its toxic effects on environment and human health [1]. In addition, lead is known as harmful to human health, especially to children, and a very small amount of lead ion could cause serious damage to the brain and central nervous system [2,3]. The United States Environmental Protection Agency (USEPA) has defined the safety limit of Pb^2+^ is 100 ppb (∼483 nM) in blood, and 15 ppb (∼72 nM) in drinking water, respectively [4,5]. Lead is the water-soluble form of lead ion and, due to its bioavailability, which can cause serious harms to human health. Therefore, the development of analytical methods for Pb^2+^ assay with high sensitivity and specificity is of considerable significance and has become a hot research topic in many fields such as environmental science, biological science, and current chemical research.

In the past decades, many analytical methods have been developed for the detection of lead ions, including atomic absorption spectrometry (AAS), [6] colormetric, [7] atomic absorption spectrometry, [8] fluorescence spectrometry, [9] and electrochemical techniques [10]. Though these methods are sensitive and accurate for Pb^2+^ assay, most of them still suffer from time-consuming, expensive, and requiring sophisticated equipment and require complicated sample pretreatment procedures. Therefore, it is remains a challenge in developing simple, sensitive, rapid, cost effective methods for detection of Pb^2+^. Recently, Surface-enhanced Raman scattering (SERS) has attracted great attention in this field due to its simplicity, low cost and high sensitivity and selectivity [11].

Surface-enhanced Raman scattering (SERS) is a physical phenomena based on the magnitude of the electromagnetic field around a noble metal surface, such as gold or silver nanoparticles [[12], [13], [14]]. In fact, SERS substrates play the critical roles in SERS method which would ensure the sensitivity and stability of the detection. In the past decades, numerous noble metal-based magnetic nanomaterials and their complexes were synthesized as high-performance SERS substrates for Raman signal enhancement [15,16]. For instance, Kim et al. reported a SERS sensing platform based on Au nanowires, enabling ultrasensitive, rapid detection of Pb^2+^ [17]. Fu et al. developed a highly sensitive and selective SERS method for determining Pb^2+^ based on a DNAzyme-immobilized plasmonic nanomachine [18]. Shi et al. Reported a SERS chips for simultaneous quantification of Pb^2+^ and Hg^2+^ based on the combination of reproducible silicon nanohybrid substrates and a corrective internal standard sensing strategy [19]. Frost et al. employed the metal-affinity properties of a citrate functionalized gold nanoparticles (AuNPs) to detect Pb^2+^ ions based on SERS [20]. Among these methods, most of them are developed by employing the DNAzyme have advantages of simple, rapid and highly sensitive for Pb^2+^ detection, but they have to suffer from the unstable and poor reproducibility of Raman signals.

DNAzyme are a kind of functional single-strand DNA or RNA which has an efficient catalytic activity on both RNAs and DNAs in vitro, [[21], [22], [23]] which have been extensively investigated due to their high metal ion specificity. In recent years, several kinds of labeled DNAzyme biosensor methods had been reported for highly sensitive detection of Zn^2+^, Hg^2+^, Pb^2+^ ions, and so on [24,25]. In 2013, Wu et al. reported the first gold nanoparticles (AuNPs) based DNAzyme sensor for detecting UO_2_^2+^ in living cells, [26] which was demonstrated to have high sensitivity. Li et al., developed a two-color AuNPs-DNAzyme fluorescence probe simultaneously tracing intracellular Zn^2+^ and Cu^2+^ [27]. Fu et al. developed a Pb^2+^ fluorescence sensor based on DNAzyme and G-quaduplex, which could be binding Pb^2+^ and result in catalytic hydrolysis of the oligonucleotide [28]. As we all know, almost all of them adopt 8–17 DNAzyme and GR-5 DNAzyme, which could recognize different specific metal ions at the same conditions [29]. However, to be the best of our knowledge, DNAzyme based-SERS aptasensor for ultrasensitive detection of metal ions has not been reported. While most of these sensors exhibit satisfying sensitivity with detection limits below the maximum contamination levels in water, an even higher sensitivity is desired if these sensors are to be used in complex biological or environmental samples.

In this work, we developed a novel kind of “turn-off’’ SERS platform, which combined AuNRs and the DNAzyme-DNA strategy together for the ultrasensitive, selective determination of Pb^2+^. Firstly, a thiolated 3′-Cy3 labeled probe (SDNA) used as the signal probe was assembled on the AuNRs through the Au-S bond. Secondly, DNAzyme hybridized with AuNRs-modified probe to produce strong SERS signal was obtained by the approaching between AuNRs and Cy3-labled probe. In the presence of Pb^2+^, the Cy3-labeled probe and AuNRs started to disassemble by catalytically cleaving of DNA molecule due to catalytic activity of DNAzyme strand (EDNA), resulting in the rupture of the substrate and produced remarkably weak SERS signals. Under the assistance of Pb^2+^, the probe in the EDNA-SDNA duplex was hydrolyzed to release DNAzyme, which could cleave another probe. In this case, the AuNRs was departed from SDNA, reducing the Raman intensity of SDNA obviously. During the reaction process, a very small number of EDNA can initiate the cleavage of many SDNA strands from AuNRs surface. The proposed strategy could detect Pb^2+^ with a broad linear range and low detection limit, In addition, we also monitor Pb^2+^ at low levels in human serum with a highly sensitive and selectively.

## 1 Experimental section

**Materials and Reagents.** Hexadecyltrimethylammonium bromide (CTAB), Gold chloride trihydrate (HAuCl_4_·3H_2_O), sodium borohydride (NaBH_4_), l-ascorbic acid (AA), P-aminothiophenol (P-ATP) and were purchased from Sigma-Aldrich (USA). 2-[4-(2-Hydroxyethyl)-1-piperazinyl] ethanesulfonic acid (HEPES) was obtained from Solarbio science&technology Co., Ltd. (Beijing, China). Oligonucleotides were designed according to the literature with an appropriate modification, [30] and synthesized and purified by Takara Biotechnology Co., Ltd. (Dalian, China). The sequences were shown below: Enzyme strand (EDNA), 5′-TCATCTCTTCTCCGAGCCGGTCGAAAGTG-3’; Substrate strand (SDNA), 5′-Cy3-CACTrAGGAAGAGATGA-SH-3’. Silver nitrate (AgNO_3_), Hg(NO_3_)_2_, CaCl_2_, FeCl_3_, CdCl_2_, BaCl_2_, MgCl_2_, KCl, CuCl_2_, ZnCl_2_, MnCl_2_, Pb(NO_3_)_2_, and Ni(NO_3_)_2_ were obtained from were obtained from Sinopharm Chemical Reagent Co., Ltd. (Beijing, China). Hydrochloric acid (HCl) was purchased from Zhongshi Chemical Co., Ltd (Shanghai, China). PBS (pH 7.4, 10 mM), fetal bovine serum (FBS), Dulbecco's modified Eagle's medium (DMEM), trypsin-EDTA, and penicillin - streptomycin were purchased from Gibco Life Technologies (AG, Switzerland). Ultrapure water (18.2 MΩ, Billerica, MA) obtained from a Milli-Q system was used through-out the study. All other chemicals were of analytical reagent grade and were used as received, unless otherwise stated. Human renal epithelial 293T cells, cervical cancer cell lines Hela cells and human hepatoma cells SMMC-7721 cells were obtained from the American Type Culture Collection (Manassas, VA). Human blood serum were provided by the Human Provincial Tumor Hospital, Central South University (China).

**Instruments.** Transmission electron microscopy (TEM) images were obtained from a JEOL-2010 instrument operated at an acceleration voltage of 200 kV. The absorption spectra were measured by a Shimadzu UV-2550 spectrophotometer (Kyoto, Japan). All Raman and SERS spectra were performed using Renishaw Invia Reflex Raman spectra were collected on a portable Raman spectrometer (B&W Tek Instruments, USA) equipped with a 785 nm laser. Surface enhanced Raman spectra were collected through 100x objective lens at the condition of 10 mW laser power and 10 s acquisition time.

**Preparation of Au Nanorods (Au NRs).** The AuNRs were synthesized according to a seed-mediated growth technique previously literature report with minor modifications [31]. Briefly, 10 mL of 0.5 mM HAuCl_4_ was mixed with 10 mL of 0.2 M hexadecyltrim-ethylammonium bromide (CTAB) solution, then added 0.6 mL of freshly prepared 0.02 M ice-cold sodium borohydride (NaBH_4_) solution into flask. The seeds were formed after stirred vigorously for 2 min and the seed solution was aged at 25 °C for 2 h.

The procedure to prepare AuNRs was as follows. First, 50 mL 0.2 M CTAB, 1 mL 10 mM AgNO_3_, 50 mL 1 mM HAuCl_4_ were mixed to prepared growth solution and left to reaction for 5 min, then added 700 μL 0.079 M ascorbic acid and left it to reduce for 2 min. The solution became colorless and 200 μL of HCl (37 wt % in water, 12.1 M) was then introduced to adjust the pH. Finally, 120 μL of gold seed solution was added to the growth solution. The mixture solution to react overnight at 30 °C in accordance with procedures reported in the literature [32,33]. The final solution was centrifuged three times at 10,000 rpm for 15 min to remove the excess CTAB. The gold nanorods were centrifuged and redispersed in 10 mL Milli-Q water.

**Preparation of Au NRs Modified SDNA.** DNA-AuNRs was prepared by modifying SDNA to the surface of AuNRs through gold-thiol bond [34]. Firstly, 50 μL of 2.5 μM thiolated SDNA (Cy3-labled probe) and 50 μL of 10 μM DNAzyme were mixed in 400 μL reaction buffer (25 mM HEPES, 0.1 M NaCl, pH = 7.04). This solution was placed in a water bath at 75 °C for 20 min and cooled down gradually to room temperature for 1 h. Released DNA probes were then separated via centrifugation at 15000 rpm for 30 min and resuspended in the 0.5 mL same buffer. Secondly, 0.5 mL EDNA-SDNA were incubated with 0.5 mL AuNRs solution suspended in 0.5 mL same buffer solution for 24 h. Then the EDNA-SDNA-AuNRs were collected by centrifugation with 7000 rpm for 30 min and resuspended in the 0.5 mL reaction buffer.

**SERS Assay of Pb**^**2+**^. For detection of Pb^2+^, different concentration of Pb^2+^ were added into as-prepared SERS platform, and incubated for 30 min at room temperature. Then the solution was centrifuged at 15000 rpm for 15 min to remove cDNA detached from dsDNA and redispersed into double distilled water. SERS detection of lead ions can be performed via disassembling of Cy3-probe@AuNRs leading to gradually decrease of Raman signals. SERS measurements were performed using a Raman spectrometer with 10 mW laser output power and the acquisition time was 10 s for all SERS spectra. Each sample measurement was performed at least for three times.

**Cell Culture.** Hela cells, 293T cells and 7404 cells were cultured in Dulbecco's modification of Eagle's medium (DMEM) supplemented with 10% Fetal Bovine Serum (FBS), 100 U/mL penicillin, and 100 μg/mL streptomycin at 37 °C in a humidified 5% CO_2_ incubator. 1 × 10^6^ cells were harvested by trypsin treatment and centrifuged at 1000g for 5 min. Cells were washed 3 times with cold PBS, centrifuged, and resuspended in 0.5 mL of ice-cold cell lysis buffer on ice for 5 min. Cells were pulse-sonicated on ice 5 times for 5 s each. Then, extracts were centrifuged by at 10000 rpm for 20 min at 4 °C, and supernatants were collected.

## 2 Results and discussion

**Scheme 1:**
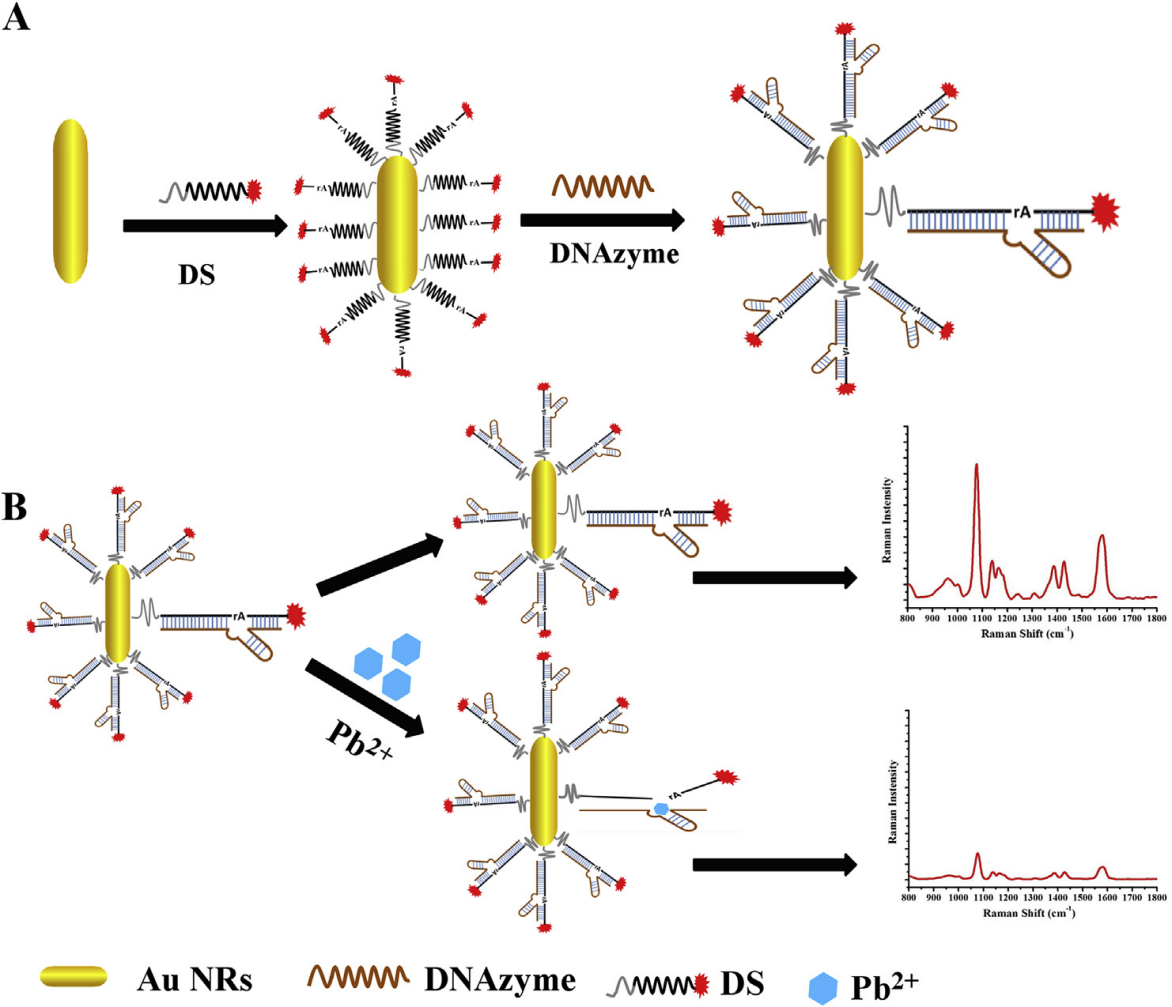
(A) Fabrication of the DNAzyme-embedded gold nanorods. (B) The principle of the SERS detection for lead ions via DNAzyme SERS biosensor.

**Working Principle of Pb**^**2+**^ **Detection.** DNAzyme (termed 17 E according to the report, which is capable of cleaving a single RNA linkage within a DNA substrate), the cleavage substrate (17 DS, a DNA/RNA chimera in which rA represents the ribonucleotide adenosine) was chosen in this study [[35], [36], [37]]. Therefore, an ideal SERS sensor based on DNAzyme should provide a sensitive response of resonance frequency by the cleavage of substrate by DNAzyme in the presence of Pb^2+^. In order to achieve the mentioned above sensing strategy, we designed a novel SERS sensor for the determination of Pb^2+^. As shown in Scheme 1A, the fabrication of SERS DNAzyme biosensor assembled by AuNR, 8–17 DNAzyme strand, and Cy3-labeled probe (ssDNA). The conjugation of AuNRs with DNAzyme and ssDNA was accomplished according to the method reported by pervious work [38]. The probe substrate with thiol group were first immobilized onto a gold-coated surface through the strong Au-S bond, which occur fluorescence resonance energy transfer between gold nanorods and fluorophore and result in remarkably SERS signal. The substrate strand hybridized with DNAzyme strand to form a DNAzyme-substrate hybrid containing a large ssDNA loop (containing 15 bases). Scheme 1B provides a detailed account of the SERS DNAzyme biosensor proposed for Pb^2+^ ion detection. Upon immobilization of enzyme strands onto the AuNRs, the substrate strands were hybridized with enzyme strands by heating and annealing. To keep an efficient hybridization between the enzyme strand and substrate strand to main the catalytic activity of the DNAzyme, the ssDNA is hybridized with enzyme strand through 10 base pairs. In the absence of Pb^2+^, DNAzyme-substrate would tightly bound to AuNRs surface and result in remarkably SERS signal. In the presence of Pb^2+^, the DNAzyme was activated and cleaved the substrate strand at the “rA” site into two parts, releasing a short Cy3-linked oligonucleotide fragment away from the AuNRs, resulted in a decreased Raman signal. The SERS intensity of Cy3-labled probe are positively related to the concentration of Pb^2+^ ions.

**Preparation and Characterization of Gold Nanorods (AuNRs) SERS Probes.** The morphologies and structure of the as-prepared AuNRs is characterized by transmission electron microscopy (TEM). Fig. 1A shows the transmission electron microscopy (TEM) image of the prepared AuNRs and we can see that the particles are fairly uniform in shape (width: 17 ± 1 nm; length: 75 ± 8 nm). AuNRs can be facilely synthesized in large scale and directly dispersed in aqueous solution without the need of surfactants or oxidation. Moreover, AuNRs were reported to allow self-assembly of thiolated compounds on its surface. On the basis of the previously established knowledge, the nanogap region between gold nanorods forms the “hot spot” region, which has the highest SERS signal. The AuNRs have a high biocompatibility, and the plasmon coupling between AuNRs lead to the stronger electromagnetic coupling and SERS effect, which the metallic nanoassemblies better applied to subsequent biological detection. As shown in Fig. 1B, US-Vis-NIR absorption spectrum of the aqueous dispersion of the nanorods was characterized. Typically, as shown in Fig. 1 C, the prominent SERS intensity of p-ATP with different concentrations (from 10^−11^ M to 10^−5^ M) adsorbed on the optimal AuNRs was measured. The characteristic peaks of p-ATP can be identified clearly even at a concentration as low as 10^−11^ M. Furthermore, we have used this SERS platform for the detection of p-ATP using AuNRs as SERS substrate to confirm the stability and reproducibility of Raman signals [39].

**Fig. 1:**
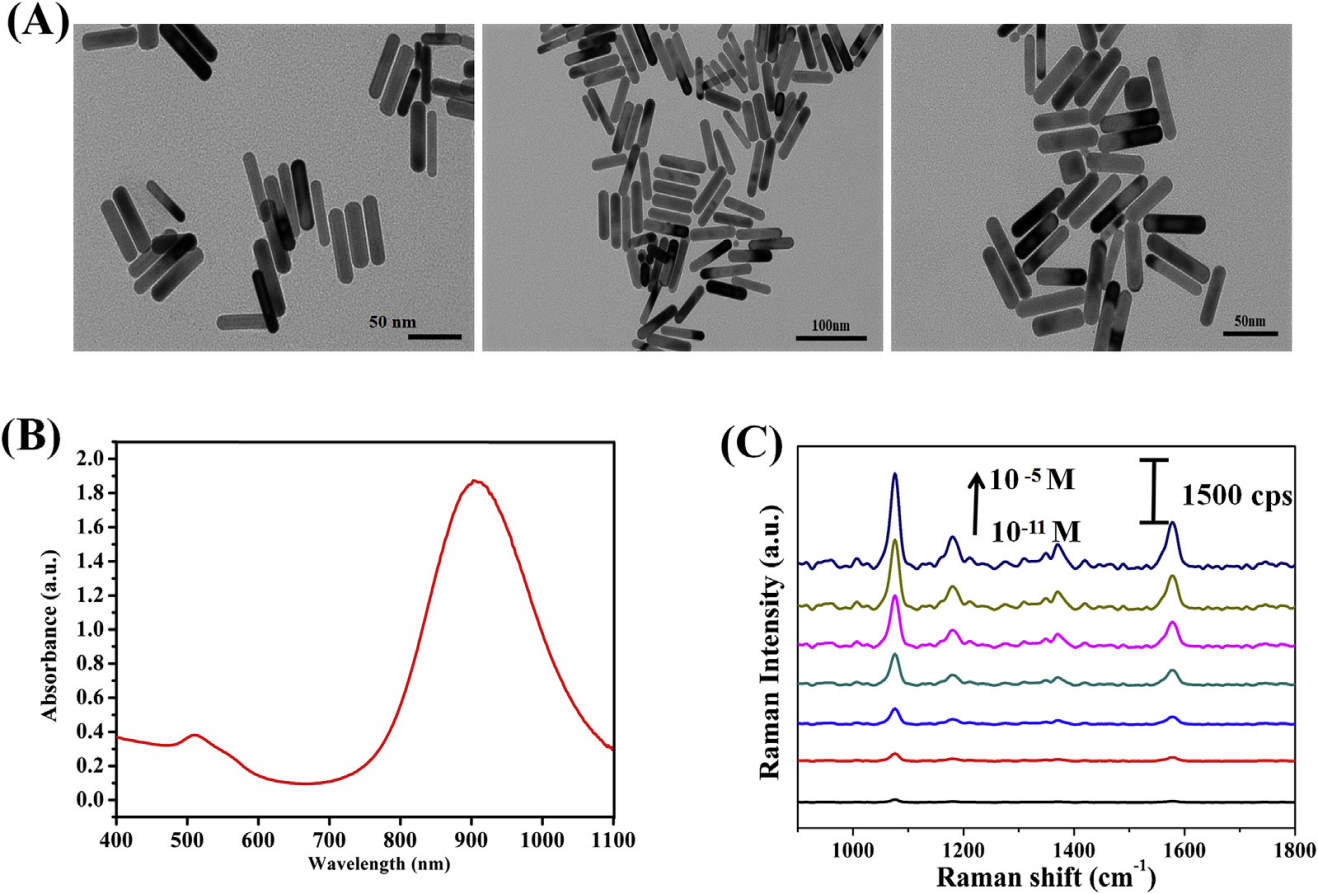
(A) TEM images of AuNRs under different magnification. (B) UV–vis absorption spectra of AuNRs solution. Inset shows the color of AuNRs solution. (C) Raman spectra of p-ATP molecules (from 10^−11^ to 10^−5^ M) from the surface of AuNRs substrate. (For interpretation of the references to color in this figure legend, the reader is referred to the Web version of this article.)

**Feasibility Test of Assay Platform.** To demonstrate the feasibility of our new sensing strategy in constructing the SERS sensing platform. As shown in Fig. 2, the Raman reporter Cy3-SDNA was assembled on AuNRs surface, which generated an obviously Raman peak at 1078 cm^−1^. While the SERS spectra of AuNRs was not obviously at the same peak (black curve). After the employ of SDNA, the SERS aptasensor achieved the “on” status. In the presence of DNAzyme strand, the SDNA-AuNRs was partly hybridized with substrate SDNA to form EDNA-SDNA duplex. It can be seen from Fig. 2 that the obviously decreased upon adding Pb^2+^ (red curve), presumably because of the SDNA-AuNRs in the EDNA-SDNA would be hydrolyzed and the EDNA was released for another probe molecule, which resulted in the far distance of AuNRs and Cy3 fluorophore.

**Fig. 2:**
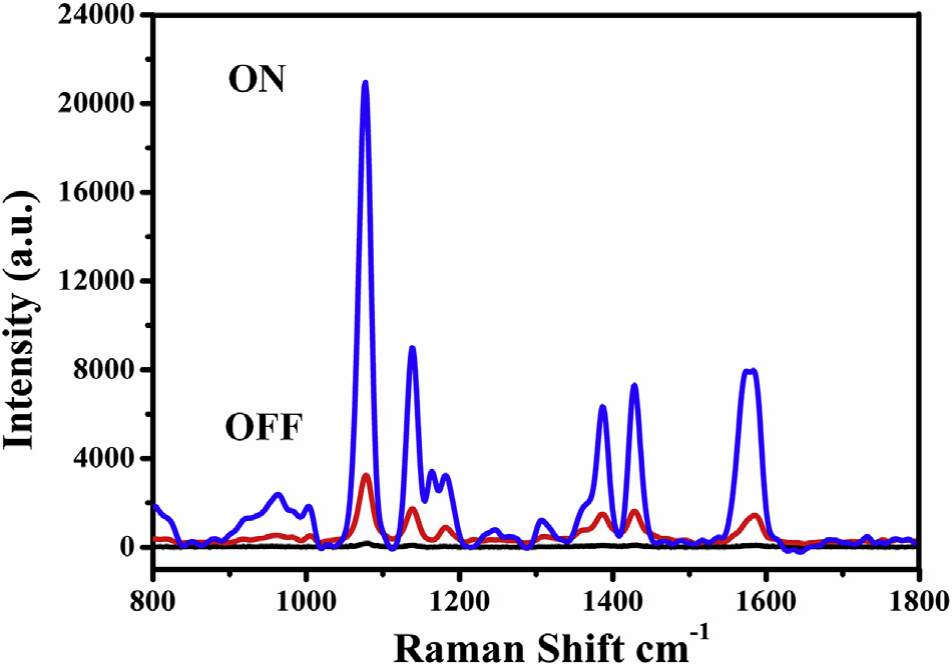
SERS platform on the state of “on” (blue curve) and “off” (red curve), and SERS spectra of AuNRs (black curve). (For interpretation of the references to color in this figure legend, the reader is referred to the Web version of this article.)

**Optimization of Assay Conditions.** In order to achieve the best sensing performance, we optimized the molar ratio of DNAzyme strand to substrate, an the incubation time in this study with 20 nM Pb^2+^ DNAzyme strand. The concentration of Cy3-labled probe is crucial factor affecting the SERS intensity of biosensor. As shown in Fig. S1A, We could see that when concentration of probe was 100 nM, the Raman intensity could reach the highest. Thus, 100 nM probe was used for the synthesis of SERS-based substrate. As shown in Fig. S1B, when the incubation time was 40 min, the Raman intensity could achieve the highest value. In this condition, the DNAzyme presented the best catalytic activity. Therefore, 40 min of incubation time were used for this strategy.

**Sensitivity Investigation.** Under the optimal assay condition, the performance of the developed strategy with different concentration of Pb^2+^ was further investigated. As shown in Fig. 3A, a dramatic decrease in the Raman intensity was observed with the increasing concentration of Pb^2+^. There are three characteristic Raman peaks in the SERS spectrum: one at 1078 cm^−1^, anther peak at 1427 cm^−1^ and the third peak at 1586 cm^−1^ [40]. In the absence of Pb^2+^, the Pb^2+^ specific DNAzyme was inactive, DNA probe could not be released form AuNRs surface, induced a remarkable strong SERS signal. Upon the addition of Pb^2+^, the DNAzyme was activated and cleaved the probe, which results in weak SERS signal at 1078 cm^−1^. Fig. 3B shows that the relationship between the SERS intensity of the 1078 cm^−1^ peak and different lead ions concentrations in logarithmic scale. A quite wide dynamic range from 0 to 100 nM was achieved for this assay. As shown in Fig. 3C, the calibration plots showed a good linear relationship between the peak of 1078 cm^−1^ and the logarithmic values of Pb^2+^ concentrations over the range from 0.5 nM to 100 nM. The linear regression equation was Y = −4631X - 855.7 with a correlation coefficient (R^2^) was 0.9973. Y in the equation is the Raman intensity of Cy3 at 1078 cm^−1^, and X is the logarithm of Pb^2+^ concentration. The limit of detection (LOD) of Pb^2+^ (S/N = 3) was estimated to be 0.01 nM, which was lower than in some other reported studies. Furthermore, the prepared SERS-active substrates are stable and still show significant SERS enhancement after two weeks of storage. It is note-worthy that such a high sensitivity was achieved in less than 40 min. The comparison of the linear range and detection limits between the proposed SERS platform and the previous other methods for detection of Pb^2+^ is summarized in Table 1. The lower detection limits might be attributed to the enormous loading of metal ions. The SERS aptasensor has some competitive advantages due to its wider linear range from and ultralow detection limit than other sensing methods in the table (Table 1).

**Fig. 3:**
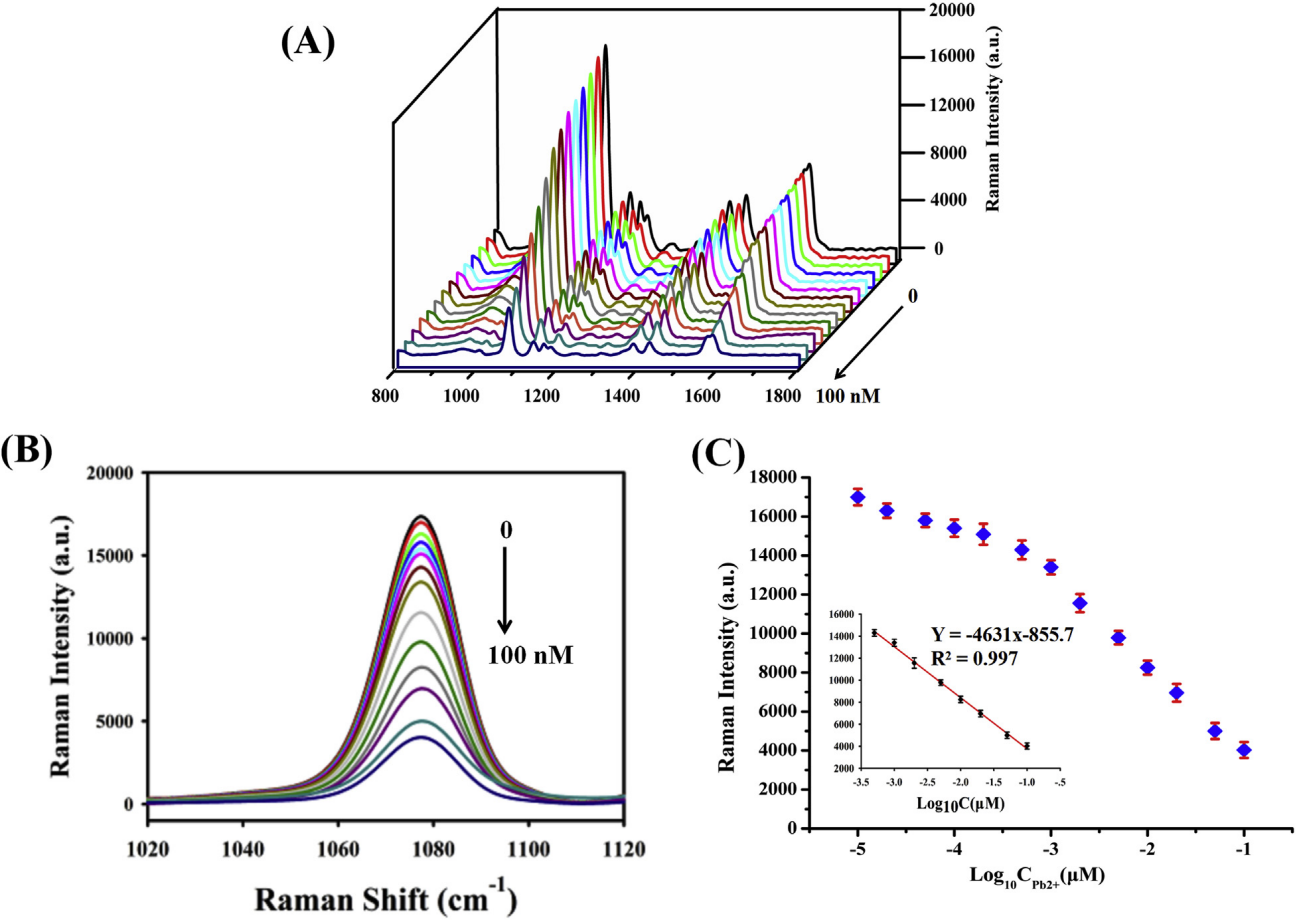
(A) SERS spectra (wave number ranging from 800 to 1800 cm^−1^) of Cy3 probe upon addition of increasing concentrations of Pb^2þ^. (B) The peak at 1078 cm^−1^ after consecutive addition of Pb^2þ^ (S/N = 3). (C) The relative intensity of the characteristic Cy3 Raman band at 1078 cm --^1^ as a function of Pb^2þ^; the inset represents the logarithmic X-scale. SERS detection parameter:λexcitation = 785 nm, accumulation time = 10 s, laser power = 100 mW.

**Table 1:** Comparison of limit of detection (LOD) and the linear range of different spectral detection methods for Pb^2+^Detection.

Detection methods	Linear range	LOD	refs
Fluorescent	5 nM to 5 μM	2 nM	[41]
Fluorescent	0 nM to 200 μM	9.64 nM	[42]
Colorimtric	3 nM to 1 μM	3 nM	[43]
Colorimetrie	25 nM to 300 μM	20 nM	[44]
electrochemistry	0.005 nM to 1000 μM	2 pM	[45]
SERS	20 nM to 1 μM	20 nM	[46]
SERS	0 nMto 100 μM	0.01 nM	this work

**Selectivity Study.** Specificity is a important factor for evaluating sensors. To investigate the specificity of the SERS sensor for Pb^2+^ detection, we selected several other metal ions, such as Ca^2+^, Co^2+^, Cu^2+^, Fe^3+^, K^+^, Mg^2+^, Mn^2+^, Ni^2+^, Pb^2+^, Zn^2+^ and the mixture have been tested. In the test, the concentration of Pb^2+^ in the solution is 50 nM and the control metal irons concentration of 10 μM, which is 200-fold higher experimental. As shown in Fig. 4A, it can be seen that weak SERS signals were observed in Pb^2+^ and mix groups compared to the other metal ions. The specific selectivity for Pb^2+^ detection is mainly attributed to the formation of coordination complexes between Pb^2+^ and DNAzyme, and would strong cleavage for Cy3-labeled probes to produce weak SERS signals. For further quantitative evaluation, corresponding Raman intensities of the 1078 cm^−1^ peak in the presence of different metal ions are presented as histograms in Fig. 4B. These results demonstrated that the proposed SERS sensor showed a good selectivity for Pb^2+^. Hence, the SERS aptasensor is considered for use in point-of -care setting recognizing Pb^2+^ and other heavy metal ions.

**Fig. 4:**
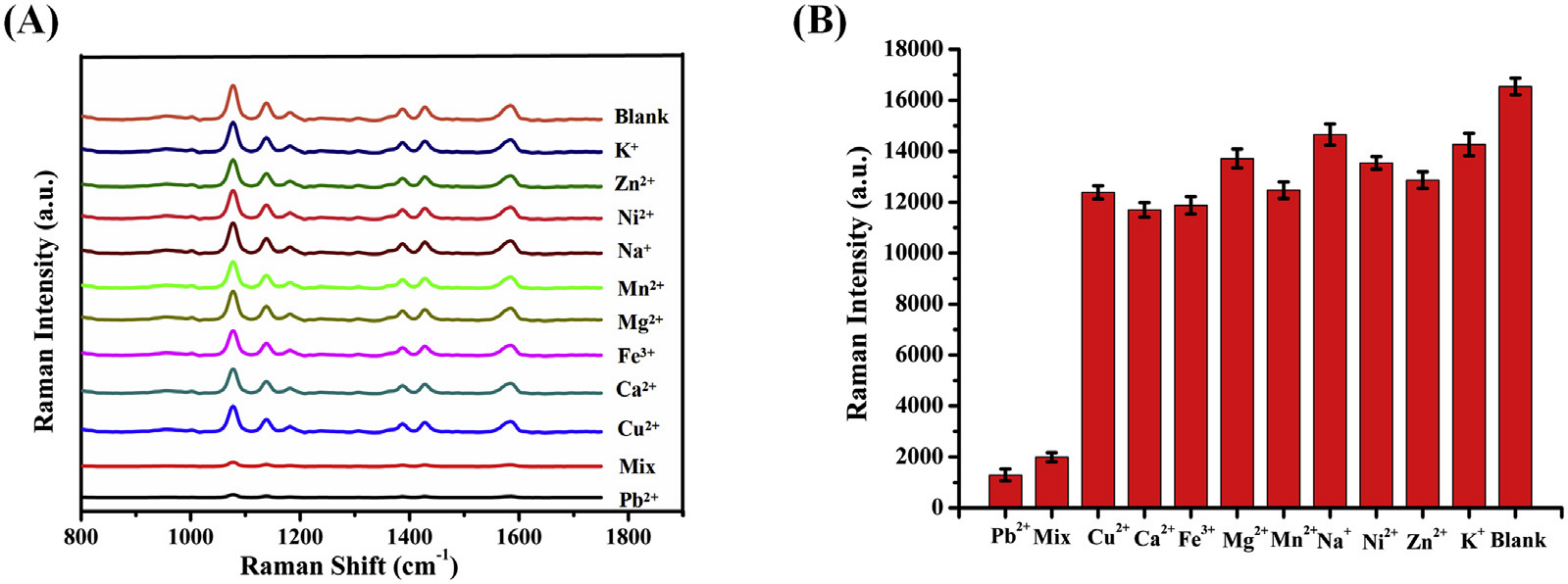
(A) SERS spectra of Pb^2+^ at ten different samples. (B) Selectivity of the SERS aptasensor toward Pb^2+^ (based on the characteristic Raman band at 1078 cm^−1^) in the presence of other metal ion species in solutions containing 10 mM of each cation for an exposure period of 40 min.

**Fig. 5:**
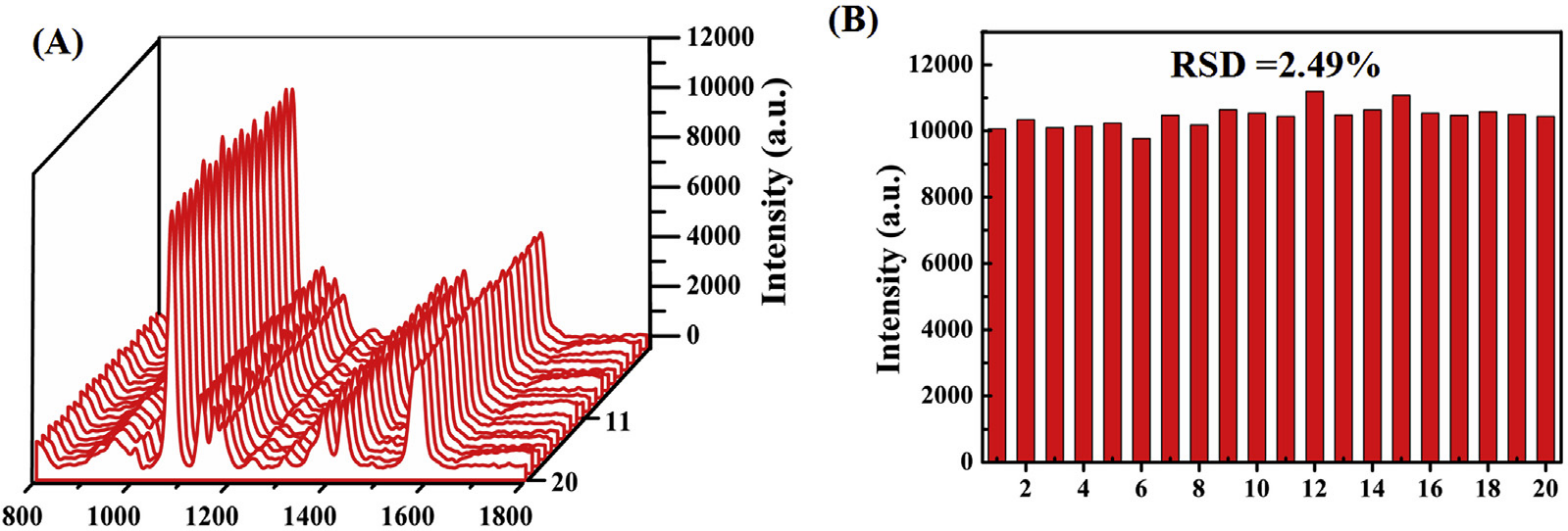
(A) SERS spectrum and (B) The Raman intensity at 1078 cm^−1^ obtained from 20 different spots. The concentration of Pb^2+^ is 2 nM.

**Reproducibility and Stability of the SERS-Based Platform.** To evaluate the reproducibility of the proposed platform, the Raman spectra of the platform of “on” status (without Pb^2+^ ion) at 20 different spots and the peak at 1078 cm^−1^ were collected to illustrate the coefficient of variation of the platform. As shown in Fig. 5A, the curves of 20 different spots were basically constant. And from Fig. 5B, the relative standard deviation (RSD) of the peak at 1078 cm^−1^ was 2.49%. These evidences proved that the proposed SERS aptasensor has good reproducibility. Stability is another advantage of this platform. To investigate the stability of SERS sensor, the AuNRs remains available for Pb^2+^ detection after it was kept at 4 °C for 2 weeks. Although the Raman intensities have evident changes, the value of I_0_/I has no obvious change when added 10 nM Pb^2+^ into reaction solution, indicating that the proposed SERS sensor has perfect storage stability over these with Raman intensity (Fig. S2).

**Fig. 6:**
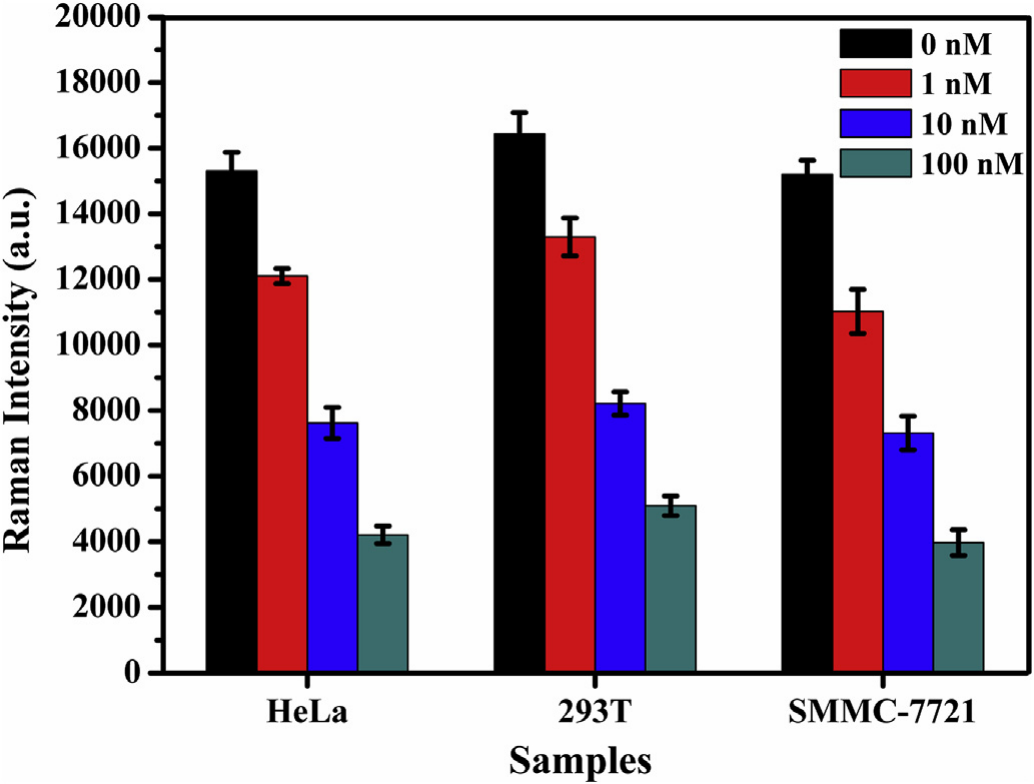
Detection of Pb^2+^ in cancer cell lysates with three different concentrations of Pb^2þ^, which were 0 nM, 1 nM, 10 nM, and 100 nM, respectively. The error bars represent the standard deviation of three repetitive experiments.

**Detection of Pb**^**2+**^ **in Real Samples.** To determine whether the novel SERS sensor platform could be applied to practical samples, which was used to perform the recoveries of three concentrations of Pb^2+^ in human serum samples. Firstly, we measured Pb^2+^ from different cell extracts including the human renal epithelial 293T cells, human hepatoma cells SMMC-7721 cells and cervical cancer cell lines Hela (Fig. 6). We also performed the novel SERS aptasensor for detection of Pb^2+^, the SERS aptasensor was carried out in real samples river water and human serum samples with three concentration levels of 5 nM, 10 nM and 100 nM, mixed it with the AuNR-conjugated aptamers and measured its resulting Raman intensity. The blood samples were centrifugated for 5 min at 3000 rpm, and then the serum supernatants were used or stored at −20 °C. Prior to the assay, the serums were 100 times diluted with PBS buffer. The analytical results are shown in Table 2. The method reveals good recovery rates of standard addition from 97.2 to 101.47%, and the relative standard deviations was less than 5.27%. The results suggested that the novel method was capable of detecting Pb^2+^ with high sensitivity and good accuracy in real complex samples.

**Table 2:** Recoveries for the detection of Pb^2+^ in real water and 1% human blood serum (n = 3).

Sample	Spiked (nM)	Detected (nM)	RSD (%)	Recovery (%)
Tap water	5	4.93	1.73	98.6
	10	9.95	2.43	99.5
	100	101.47	4.33	101.47
River water	5	4.86	3.75	97.2
	10	10.02	2.10	100.2
	100	96.81	3.19	96.81
Human serum	5	5.03	2.41	100.6
	10	10.13	5.27	101.3
	100	100.73	4.13	100.73

## 3 Conclusion

In summary, a novel, facile and sensitive SERS aptasensor was developed for Pb^2+^ detection using SH-SDNA conjugated with AuNRs as a substrate and Pb^2+^-specific DNAzyme to capture lead ions. To the best of our knowledge, the sensitivity of this assay for the detection of Pb^2+^ is the lowest among the aptamer-based sensors that have been developed. This SERS platform showed a wide linear range (0–100 nM), a low detection limit (0.01 nM), and an acceptable stability, selectivity and reproducibility. In addition, the present sensor could be applied to detect Pb^2+^ in water and human serum samples, respectively. On the whole, it can be expected that this SERS aptasensors may offer a new method for sensitive and selective detection of other metal ions and molecules.

## Figures and Tables

**figs1:**
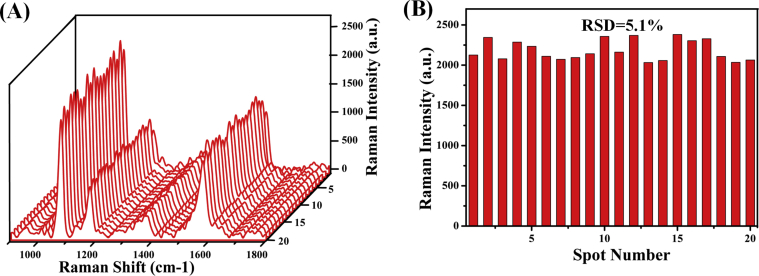


**figs2:**
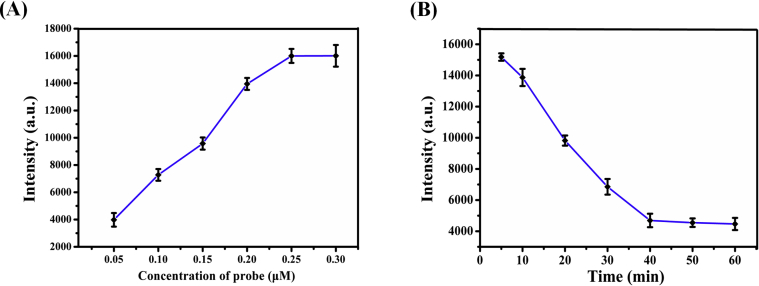


**figs3:**
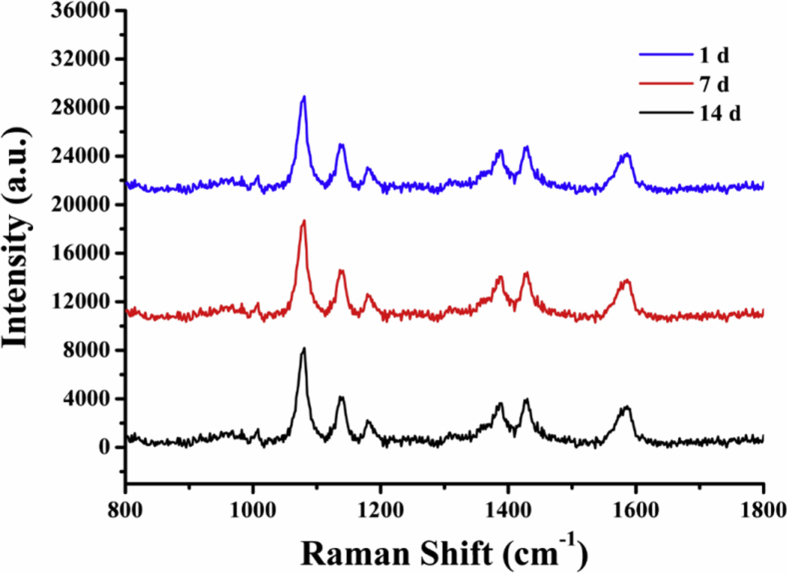


**figs4:**
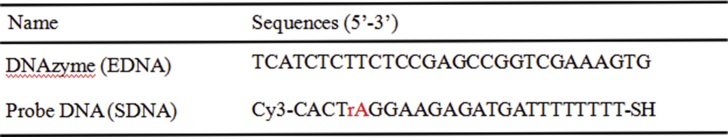


**figs5:**